# A study of validity and usability evidence for non-technical skills assessment tools in simulated adult resuscitation scenarios

**DOI:** 10.1186/s12909-023-04108-4

**Published:** 2023-03-11

**Authors:** Helen Higham, Paul Greig, Nick Crabtree, George Hadjipavlou, Duncan Young, Charles Vincent

**Affiliations:** 1 Nuffield Department of Clinical Neurosciences, University of Oxford, Level 6, West Wing, John Radcliffe Hospital, Oxford, OX3 9DU England; 2 The Medical Specialist Group LLP Guernsey, Saint Peter Port, Guernsey; 3 Oxford University Hospitals NHS Foundation Trust, Oxford, England; 4 Department of Experimental Psychology, University of Oxford, Oxford, UK

**Keywords:** Nontechnical skills assessment, Simulation-based education, Validity, Internal consistency, Interrater reliability, Response process, Usability

## Abstract

**Background:**

Non-technical skills (NTS) assessment tools are widely used to provide formative and summative assessment for healthcare professionals and there are now many of them. This study has examined three different tools designed for similar settings and gathered evidence to test their validity and usability.

**Methods:**

Three NTS assessment tools designed for use in the UK were used by three experienced faculty to review standardized videos of simulated cardiac arrest scenarios: ANTS (Anesthetists’ Non-Technical Skills), Oxford NOTECHS (Oxford NOn-TECHnical Skills) and OSCAR (Observational Skill based Clinical Assessment tool for Resuscitation). Internal consistency, interrater reliability and quantitative and qualitative analysis of usability were analyzed for each tool.

**Results:**

Internal consistency and interrater reliability (IRR) varied considerably for the three tools across NTS categories and elements. Intraclass correlation scores of three expert raters ranged from poor (task management in ANTS [0.26] and situation awareness (SA) in Oxford NOTECHS [0.34]) to very good (problem solving in Oxford NOTECHS [0.81] and cooperation [0.84] and SA [0.87] in OSCAR). Furthermore, different statistical tests of IRR produced different results for each tool. Quantitative and qualitative examination of usability also revealed challenges in using each tool.

**Conclusions:**

The lack of standardization of NTS assessment tools and training in their use is unhelpful for healthcare educators and students. Educators require ongoing support in the use of NTS assessment tools for the evaluation of individual healthcare professionals or healthcare teams. Summative or high-stakes examinations using NTS assessment tools should be undertaken with at least two assessors to provide consensus scoring. In light of the renewed focus on simulation as an educational tool to support and enhance training recovery in the aftermath of COVID-19, it is even more important that assessment of these vital skills is standardized, simplified and supported with adequate training.

**Supplementary Information:**

The online version contains supplementary material available at 10.1186/s12909-023-04108-4.

## Background

Safe care of acutely unwell patients in dynamic clinical settings, such as the operating theatre or emergency department requires high levels of competency in both technical and non-technical skills (NTS).

The use of experiential learning [1] incorporating immersive simulation can enhance competence in NTS (including situation awareness (SA) and communication) and improve patient outcomes [2, 3]. Simulation training in healthcare has expanded over the past two decades, and healthcare professionals are now trained, revalidated and assessed in simulated scenarios. Tools designed to assess NTS must have adequate evidence to support their validity, and they must be used by educators who are trained to do so.

In recent years questions have arisen around the use of “non-technical skills” as a term to describe these important individual and team behaviors in healthcare [4, 5], but in the absence of formal consensus we will use NTS for this study.

Assessments of NTS for individuals or teams requires: understanding of the characteristics of NTS; appreciation of the overt behaviors which are exemplars for a particular NTS (e.g. clear communication of mental models for good SA) and calibration of assessors. These are not intuitive skills, and training is required to use NTS instruments reliably. The Civil Aviation Authority clearly describes what is expected of its examiners, and mandates regular training and revalidation for assessors in the use of behavioral rating systems [6]. A recent expert panel put forward similar recommendations for training healthcare professionals in the use of NTS assessment tools [7], but as yet there is no such requirement for clinical educators. We have previously highlighted the wide variation in clinical settings, applicability, and evidence of validity (including internal structure, response process and relations with other variables – see below) for 76 NTS assessment tools in healthcare, which poses a significant challenge for educators in choosing the most appropriate one to use [8].

We used a modern framework to consider validity evidence [9] for three NTS assessment tools designed in the UK. This framework was chosen as it has unified and simplified previous frameworks and has been adopted by international bodies responsible for medical education. It requires evidence from five sources: content (evidence that the assessment tool is measuring what it is intended to measure), internal structure (this is usually described as evidence of reproducibility across elements of the assessment tool), response process (describes how well assessor or participant actions align with the intended attribute), relations with other variables (describes statistical associations of an assessment tool with another tool that has a particular theoretical relationship) and consequences (decisions or actions which result from the assessment). We have excluded evidence for content validity because this has been extensively discussed in the original papers [10–12]. We did not consider consequences evidence because we were not able to analyze the impact of the assessment.

The aims of the study were to enhance the understanding of the limitations of specific NTS assessment tools, and what key features might be considered by educators before choosing one. We did this by:

- Assessing validity evidence for:
  - ◦ internal structure (internal consistency and inter-rater reliability) of the Anesthetists’ Non-Technical Skills tool (ANTS) [13], the Observational Skill based Clinical Assessment tool for Resuscitation (OSCAR) [11] and the revised Oxford Non-Technical Skills tool (Oxford NOTECHS) [14]
  - ◦ response process
  - ◦ relations with other variables
- Analyzing the usability of ANTS, OSCAR, and Oxford NOTECHS

## Methods

### Study design and ethics approval

Mixed quantitative and qualitative methods were used to undertake a secondary analysis of standardized videos recorded during a study to investigate the value of simulation training for anesthetists in their first year of specialist training (the ST1 Anesthetic Recruit Training [START] study). Original ethical approval (including acquiring informed consent from all participants) for the videos was obtained via the Central University Research and Ethics Committee (ref: MSD/IDREC/C1/2011/137). This follow-up study protocol was submitted to the University of Oxford’s Institutional Review Board (the Clinical Trials and Research Governance Committee) and was accepted as a secondary review within the original terms of consent requiring no further ethical approval. Ten videos were selected randomly from a pool of 50 adult life support (ALS) scenarios. We used a standardized adult acute severe asthma scenario in which the manikin develops a tension pneumothorax and deteriorates to the point of cardiac arrest (pulseless electrical activity – PEA). Scenarios lasted an average of 14 min 5 s and involved a trainee anesthetist, and two faculty members in the roles of nursing assistants.

### Participants and procedures

Three Consultant Anesthetists (Attending Anesthesiologists) with greater than 10 years’ experience in simulation-based education and trained in the use of the ANTS tool were involved in the study because there is evidence that greater clinical experience improves inter-rater reliability [15]. The ten ALS scenarios were reviewed alone by each rater, and the participants’ NTS rated using ANTS, OSCAR, and Oxford NOTECHS. Random numbers were assigned to the videos so that they were viewed in a different order each time for each tool. All video analyses were undertaken in environments optimized for uninterrupted viewing. Score sheets for each tool were marked by hand (see Additional file 1) and data were transcribed into a spreadsheet for subsequent analysis. Data were anonymized and stored securely.

### NTS assessment tool selection

Several authors have highlighted the importance of the culture in which a tool for the measurement of NTS is to be used [16–21]. Therefore, three tools which had originally been developed and validated in the UK for staff in the NHS were chosen. ANTS, OSCAR and Oxford NOTECHS displayed considerable variability in original study design, context of use and data analysis and a summary of these differences is provided in Table 1.

**Table 1:** Variation in original methods, data collected and statistical analysis for ANTS, Oxford NOTECHS and OSCAR

NTS assessment tool	Original method of testing tool	Data assessed	Statistical tests used
**ANTS – for assessment of an anesthetist only**	50 anesthesiologists trained, new to NTS rating, each rated 8, non-standardized videos of simulated anesthetic scenarios	All elements [15] and category [4] scores, no global score.	Accuracy of scores with percent agreement (± 1 scale point) to reference ratings and mean absolute difference IRR with r_WG_
**Oxford NOTECHS - for assessment of three theatre teams – surgical, anesthesia and nursing**	6 assessors (3 clinical, 3 human factors experts) assessed a total of 297 live surgical procedures in pairs (surgeon and human factors expert)	Total scores for categories in sub-teams and overall summated score (for all categories and all teams)	Differences in mean scores, IRR with ICC for global and category scores
**OSCAR - for assessment of a resuscitation team in the following groups: anesthesia, physician and nursing groups**	2 clinical expert assessors, each rated 8, non-standardized videos of simulated cardiac arrest scenarios	Scores for each element, overall category score and a summated global score	Descriptive statistics to explore mean scores between raters, IRR with ICC for global and category scores

The authors of OSCAR and Oxford NOTECHS were contacted as our study would be assessing only one of the teams (i.e., the physician team in OSCAR [physician in this case refers to the doctor leading the arrest team which was an anesthetist in this study] and the anesthesia team in Oxford NOTECHS) described in their systems, and our approach was approved as an acceptable use of the tools.

### Internal structure

Reliability of the assessment tools was analyzed using Cronbach’s alpha for internal consistency across all raters for global scores and category scores in each tool. The statistical tests used to calculate interrater reliability (IRR) were those commonly used for the purpose in similar assessment tools [8]: weighted (Cohen’s) kappa, Intraclass Correlation Coefficients (ICC), and within groups reliability scores (r_WG_).

The weighted kappa can only be used to compare two raters at a time, therefore, we randomly allocated one pair for this analysis (Raters 1 and 3) and calculated ICC and r_WG_ for all three raters and Raters 1 and 3 alone.

When the within groups reliability score (r_WG_) was applied to our data it revealed very high levels of agreement in all categories for all raters (i.e., it did not discriminate at all between raters). We excluded r_WG_ from the analysis as it is subject to significant test bias and benchmarking is only possible for tests using a score range equal to or greater than five with 10 raters or more. Interrater reliability was, therefore, calculated with ICC (average-measures) and weighted kappa only for the overall NTS tool scores and for each of the NTS categories.

### Response process: scoring systems for ANTS, Oxford NOTECHS and OSCAR

ANTS, Oxford NOTECHS and OSCAR divide NTS into different categories and elements (we have chosen the ANTS taxonomy here for simplicity and consistency), score individuals or teams and use different scoring systems (see Additional file 1). To compare the scales, it was necessary to standardize the way in which we assessed our data at the element and category level. A summated score of the categories in ANTS was added (as this is normal practice for Oxford NOTECHS and OSCAR) and element scores were recorded for Oxford NOTECHS as this is normal practice for ANTS and OSCAR. A comparison is provided in Table 2. Scores for Oxford NOTECHS were only recorded for the anesthesia team as there was no surgical or nursing team in the scenario and, similarly, for OSCAR only a physician team score was recorded (in this study the physician leading the arrest team was the anesthetist) as there was no anesthesia or nursing team.

**Table 2:** Differences in structure and scoring for ANTS, Oxford NOTECHS and OSCAR

System and profession(s) assessed	Categories	Number of elements	Score
**ANTS** Rating for anesthetist only	Task management	4	1–4 (plus “not observed”) for elements, overall category score No global score for all categories **Summated global category scores added** (20 scores per video)
	Team working	5	
	Situation awareness	3	
	Decision making	3	
	Total = 4	Total = 15	
**Oxford NOTECHS** Rating for three theatre teams – surgical [S], anesthetic [A] and nursing [N], all use the same categories and elements	Leadership and management (S,A,N)	5	1–8 for categories Includes global summated score for categories **Score for each element added** (21 scores per video)
	Teamwork and co-operation (S,A,N)	4	
	Problem solving and decision making (S,A,N)	4	
	Situation awareness (S,A,N)	3	
	Total = 4	Total = 16	
**OSCAR** Rating applied to resuscitation team in the following groups: anesthetic group [A], physician group [P] and nursing group [N], elements specific to each group	Communication	A = 4,*P* = 3,*N* = 3	0–6 for elements and categories Includes global summated score for categories (25 scores per video)
	Co-operation	A = 2,*P* = 2,*N* = 3	
	Co-ordination	A = 2,*P* = 2,*N* = 3	
	Leadership	A = 3,*P* = 3,*N* = 2	
	Monitoring (SA)	A = 3.*P* = 3,*N* = 2	
	Decision making	A = 3,*P* = 2,*N* = 3	
	Total = 6	Total = 32	

Adaptations for this study are highlighted in bold font and underlined

### Relations with other variables

Relations with other variables was measured by calculating Pearson correlation coefficients using normalized global rating scores for each rater with each tool.

### Usability of ANTS, Oxford NOTECHS and OSCAR

#### Training to use NTS assessment tools

All three raters have received formal training in the use of the ANTS system. Training in the use of Oxford NOTECHS and OSCAR was devised through discussion with the tools’ authors and providing them with an explanation of the experience of the investigators and the design of the study. To ensure commonality of approach, the three raters read the materials provided and then reviewed five randomly assigned ALS videos together using ANTS, OSCAR and Oxford NOTECHS with discussion of scoring differences and the nuances of use of the tools.

Quantitative and qualitative assessments of the ease of use of each tool were made.

#### Quantitative measures of usability

- Time taken to train to use the assessment tools (including reading and assimilating Information; meeting to assure consensus on use of the tools; and a group training and familiarization session)
- Completeness of data points filled for each system
- Time taken to review and score the videos using each assessment tool (measured for Rater 1 only as Raters 2 and 3 had been involved in original review of the START videos)
- Quantitative data from the usability questionnaire (see below)

#### Qualitative measures of usability

- Questionnaire adapted from the usability assessment for the development of the ANTS-AP NTS [22] assessment system (Additional file 2). Questionnaires were answered independently by each investigator at the training session and then again after review of the ten study videos
- Post-study focus group to discuss tool attributes

### Data analysis

All statistical tests were undertaken using SPSS® (IBM®V27.0). Scores for each system (global and category) were assessed for normality of distribution and are displayed as raw and percentage scores. Comparisons of descriptive statistics were made between global scores for each system (i.e., all categories combined) and between the SA categories, as all the assessment tools used three elements to score SA (none of the other categories had the same number of elements contributing to the score). Scores were also analyzed for floor or ceiling effect.

(8)Time taken to assess videos using each tool was compared using one-way ANOVA.

## Results

### Scores using ANTS, Oxford NOTECHS and OSCAR

Global scores were normally distributed (Shapiro-Wilk > 0.05), except for Rater 2 with ANTS, but category scores were not normally distributed (Shapiro-Wilk < 0.05) therefore, Table 3 shows the median, range and interquartile range (IQR) for global and category scores for each rater using each system.

**Table 3:** Median raw scores (global [summated category] scores and for each category) for ANTS, Oxford NOTECHS and OSCAR

Scoring system and NTS category	Rater 1 Median (range) [IQR]	Rater 2 Median (range) [IQR]	Rater 3 Median (range) [IQR]
**ANTS (scale 1–4)**			
Global score (all categories)	12.5 (8–16) [4]	16.0 (14–16) [1]	12.0 (9–14) [3]
Task management	3.0 (2–4)[0]	4.0 (4, 4)[0]	3.0 (2–3) [1]
Teamwork	3.5 (2–4) [1]	4.0 (3–4)[0]	3.5 (2–4) [2]
Situation Awareness	3.0 (2–4)[0]	4.0 (3–4)[0]	3.0 (1–3)[0]
Decision making	3.0 (2–4) [1]	4.0 (3–4)[0]	3.0 (3–4)[0]
**Oxford NOTECHS (scale 1–8)**			
Global score (all categories)	27.0 (16–31) [7]	28.5 (22–32) [7]	25.0 (16–29) [6]
Leadership and management	7.0 (5–8) [2]	7.0 (5–8) [2]	6.0 (4–7) [2]
Teamwork and cooperation	6.5 (4–8) [2]	7.0 (6–8) [2]	6.0 (3–8) [2]
Problem solving and decision making	6.5 (3–7) [2]	7.0 (6–8) [2]	6.0 (4–7) [1]
Situation Awareness	7.0 (4–8) [1]	6.5 (5–8) [2]	6.0 (5–7) [1]
**OSCAR (scale 0–6)**			
Global score (all categories)	31.0 (20–34) [10]	26.5 (23–36) [11]	25.5 (13–34) [9]
Communication	5.0 (4–6) [1]	4.5 (3–6) [1]	4.0 (2–6) [2]
Cooperation	5.0 (3–6) [2]	4.0 (3–6) [1]	4.0 (2–5) [2]
Coordination	5.0 (4–6) [1]	4.0 (4–6) [2]	4.0 (2–5) [2]
Leadership	5.0 (3–6) [1]	4.5 (4–6) [2]	4.0 (2–6) [1]
Situation Awareness	5.0 (3–6) [2]	4.5 (4–6) [2]	4.5 (3–6) [1]
Decision making	5.0 (3–6) [2]	5.0 (4–6) [2]	4.0 (3–6) [1]

*IQR* interquartile range, score ranges for each system are shown in parenthesis

**Fig. 1:**
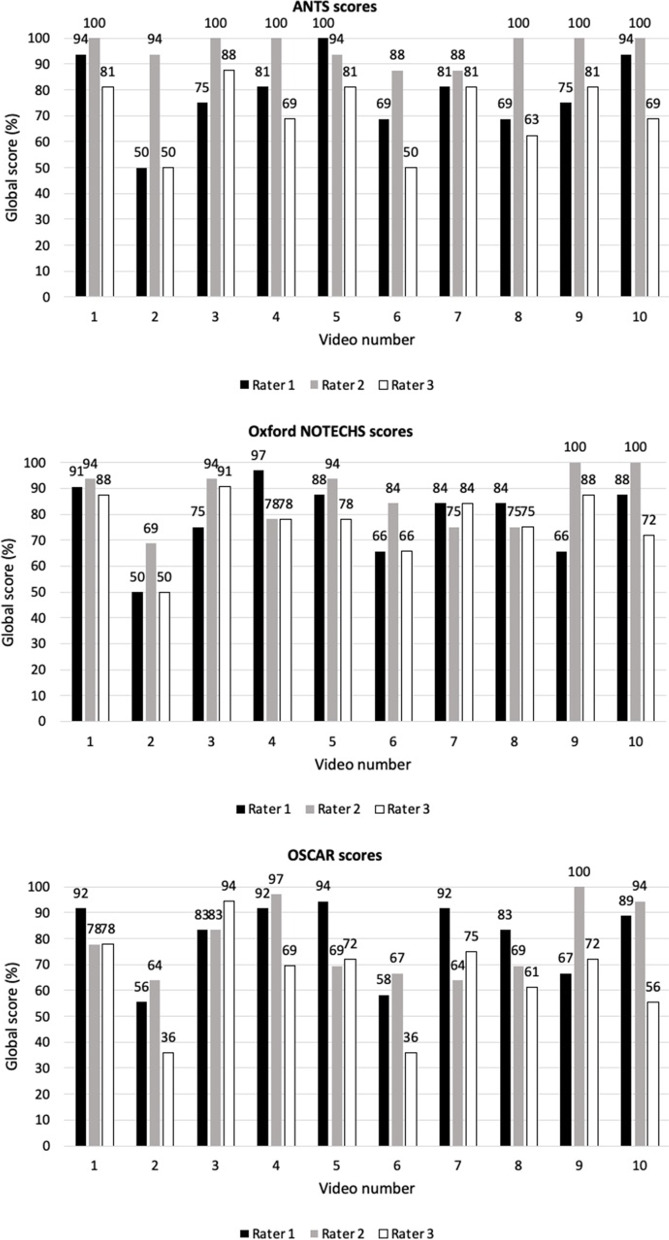
Percentage global scores for each video scored independently using ANTS, Oxford NOTECHS and OSCAR

Median scores for performances were above average for all raters and all systems suggesting that the individual anaesthetist (ANTS) and the teams (Oxford NOTECHS and OSCAR) in the videos were generally performing well. Median ANTS scores for Rater 2 were a maximum four points suggesting a ceiling effect was evident for this rater with this tool. Percentage scores were calculated to allow comparison across the different assessment tools (see Table 4 and Fig. 1). The “conflict solving” element of the teamwork and cooperation category for Oxford NOTECHS was not relevant in the context of the ALS scenario and so was removed from the analysis.

**Table 4:** Percentage global (summated category) and individual category scores for ANTS, Oxford NOTECHS and OSCAR for each rater

Scoring system and NTS category	Rater 1 (%)	Rater 2 (%)	Rater 3 (%)
**ANTS**			
Global score (all categories)	78	100	75
Task management	75	100	75
Teamwork	87.5	100	87.5
Situation Awareness	75	100	75
Decision making	75	100	75
**Oxford NOTECHS**			
Global score (all categories)	84	89	78
Leadership and management	87.5	87.5	75
Teamwork and cooperation	81	87.5	75
Problem solving and decision making	81	87.5	75
Situation Awareness	87.5	81	75
**OSCAR**			
Global score (all categories)	74	63	61
Communication	71	64	57
Cooperation	71	57	57
Coordination	71	57	57
Leadership	71	64	57
Situation Awareness	71	64	64
Decision making	71	71	57

Percentage scores revealed differences between raters and assessment tools but global scores for OSCAR were the lowest for all raters. Figure 1 provides a breakdown of scores for each video and each assessment tool. The lowest scoring video when scores were averaged across raters was video two.

### Internal structure evidence

#### Internal consistency

Cronbach’s alpha was used to calculate internal consistency for all raters using all assessment tools (see Table 5). Results were good (> 0.7 is considered satisfactory [23]) across all categories combined (global scores). Scores are highlighted in individual categories where they fall below 0.7 and this happened for Rater 2 and Rater 3 mainly for ANTS, the most familiar system.

**Table 5:** Cronbach’s alpha for scores from each rater for ANTS, Oxford NOTECHS and OSCAR

Rater	1	2	3
**ANTS**			
All categories (global)	0.95	0.80	0.92
Task management	0.71	**0.20**	**0.51**
Teamwork	0.90	0.87	0.93
Situation Awareness	0.90	**0.60**	0.70
Decision making	0.70	**0.20**	**0.54**
**Oxford NOTECHS**			
All categories (global)	0.99	0.96	0.97
Leadership and management	0.96	0.89	0.93
Teamwork and cooperation	0.95	0.89	0.95
Problem solving, decision making	0.96	0.78	0.94
Situation Awareness	0.97	0.94	0.72
**OSCAR**			
All categories (global)	0.97	0.97	0.98
Communication	0.86	0.88	0.82
Cooperation	0.77	0.77	**0.68**
Coordination	0.80	0.89	0.96
Leadership	0.85	0.93	0.89
Situation Awareness	0.91	0.84	0.91
Decision making	0.91	0.86	0.96

Scores are highlighted in bold and underlined where they fall below the acceptable level of reliability for summative settings

#### Inter-rater reliability

Inter-rater reliability was calculated in SPSS with ICC (using a consistency definition) and weighted kappa and results comparing the three investigators using each tool are shown in Table 6.

**Table 6:** IRR results for all raters or paired raters (Raters 1 + 3)) using ICC, and weighted kappa. The two raters used for the weighted kappa analysis were chosen randomly because weighted kappa can only compare 2 raters at a time

Scoring system and NTS category	ICC: Raters 1 + 3	ICC: all three raters	Weighted kappa (Raters 1 + 3 only)
**ANTS**			
Global score	**0.73**	0.62	0.52
Task Management	0.26	N/A^a^	0.10
Teamwork	0.65	0.62	**0.64**
Situation Awareness	**0.79**	0.60	0.54
Decision Making	0.64	0.67	0.45
**Oxford NOTECHS**			
Global score	**0.71**	0.69	0.54
Leadership and Management	0.46	0.50	0.28
Teamwork and Cooperation	**0.77**	**0.76**	**0.61**
Problem Solving and Decision Making	**0.81**	0.67	**0.67**
Situation Awareness	0.34	0.51	0.22
**OSCAR**			
Global score	**0.80**	0.68	0.40
Communication	0.53	0.25	0.25
Cooperation	**0.84**	0.69	0.54
Coordination	**0.72**	**0.75**	0.26
Leadership	**0.75**	0.67	0.29
Situation Awareness (monitoring)	**0.87**	0.67	**0.73**
Decision making	0.64	0.66	0.41

Scores are highlighted (bold and underlined) where good or better agreement occurred and underlined where *p* = < 0.05

^a^The comparison between 3 raters was not possible for the Task Management domain of ANTS because the scores for rater 2 had zero variance

The ICC results show good or better agreement using benchmarking described by Downing (a score > 0.7 represents good agreement, but higher scores would be required for high stakes settings) [24] when Raters 1 and 3 are compared for all global scores and for most categories in OSCAR, the teamwork and problem solving categories in Oxford NOTECHS and the SA category for ANTS.

Altman’s [25] updated version of the Landis and Koch [26] benchmarking system was used to judge results for the kappa statistic results (a score > 0.6 indicates good agreement). Good agreement was only observed in the teamwork category for ANTS, the teamwork and cooperation and problem solving and decision making categories for Oxford NOTECHS and the SA domain for OSCAR.

### Response process

Scoring systems for each of the tools were different (see above) and a ceiling effect for Rater 2 was observed for ANTS. Analysis of raters thoughts and actions is also evidence of validity and is described in the section on usability below. Time taken for scoring was significantly lower for ANTS (see below). The OSCAR scoring system was found to be most difficult to use and raters also found it the least flexible. The scoring sheets were a problem for two of the three raters in Oxford NOTECHS and all three found the scoresheet for OSCAR difficult to use (see below).

### Relations with other variables

Pearson correlation coefficients were calculated for global scores (as percentages) for each rater using each tool. Preliminary analyses showed relationships to be linear for each assessment tool (with no outliers), and variables from NOTECHS and OSCAR (but not ANTS) were normally distributed, as assessed by Shapiro-Wilk’s test *p* > 0.05. It was, therefore, decided to proceed with a Pearson’s correlation test as it is somewhat robust to non-normal data. Results are shown in Table 7.

**Table 7:** Pearson correlation coefficients with *p* values for each rater using all three tools

Rater	Correlation Coefficients for NTS Tools *r* (p value)		
	ANTS / NOTECHS	ANTS / OSCAR	NOTECHS / OSCAR
1	0.81** (0.005)	0.86** (0.001)	0.91** (< 0.001)
2	0.58 (0.08)	0.65* (0.04)	0.72* (0.02)
3	0.91** (< 0.001)	0.95** (< 0.001)	0.95** (< 0.001)

Significance level ** = *p* < 0.01 * = *p* < 0.05

There were statistically significant positive correlations for raters 1 and 3 (at a significance level or *p* = < 0.01) with all three tools and for Rater 2 (at a significance level of *p* = < 0.05), with ANTS with OSCAR and Oxford NOTECHS with OSCAR.

### Usability measures for ANTS, Oxford NOTECHS and OSCAR

#### Quantitative measures: training time

The initial period of familiarization with the two tools which had not previously been used by the investigators comprised 3 h reading the original papers for OSCAR and Oxford NOTECHS and reviewing their scoring systems followed by a four-hour session of video reviews and discussion using ANTS, Oxford NOTECHS and OSCAR as described above.

#### Quantitative measures: time taken to score videos

Mean time in minutes (95% CI) to complete scoring of the videos by Rater 1 was 15.3 (13.8–16.7) for ANTS, 18.5 (16.6–20.5) for OSCAR and 19.6 (17.7–21.4) for Oxford NOTECHS (times include the length of the video. Data were complete on all score sheets for all systems and all raters.

The one-way ANOVA test (including a post hoc Tukey test) was applied to compare times taken to use each of the assessment tools and revealed that time taken to use ANTS was significantly lower than Oxford NOTECHS (*p* = 0.02) and OSCAR (*p* = 0.002) but there was no significant difference between Oxford NOTECHS and OSCAR.

#### Quantitative measures: usability questionnaire

OSCAR scored lowest across questions relating to behaviors described (questions 3,4,5,9,15) and ease of use (questions 6,7,10,11,12) when compared with ANTS and Oxford NOTECHS (see Additional file 3). One rater felt that more information for training to use OSCAR was necessary. The final question [16] on overall usability was also negative from all raters for OSCAR.

#### Qualitative measures: usability questionnaire

Qualitative data from the usability questionnaires and the subsequent review meeting are summarized here with quotes taken from written or verbal transcripts (see Additional file 3).

Comments about the systems overall highlighted the differences in context of use: ANTS “can only be used to score the anesthetist in the team” whereas Oxford NOTECHS and OSCAR “assess three sub-teams” although it was highlighted that this would require additional context specific expertise from the faculty. Oxford NOTECHS was found to be easier to use than OSCAR because of the similarity of construct to ANTS.

The rating scales for Oxford NOTECHS and OSCAR were preferred to ANTS because it was felt less likely that a ceiling effect would be observed. However, one sided assessment sheets were preferred and OSCAR’s three page layout was considered unwieldy.

## Discussion

This study has explored the evidence of validity and usability for three different tools for NTS assessment in the context of a standardized simulated emergency scenario. A similar study considered three different tools [27] (TEAM [28], T-NOTECHS [29] and TTCA [27]), and three raters assessed 10 non-standardized videos of real trauma care episodes (five emergency and five non-emergency) using the tools. The three raters trained to use the less familiar tools (TEAM and T-NOTECHS), similarly to this study, found a variation in IRR (using ICC) which resonated with our findings. The results of our study highlighted additional issues in internal structure, response process, relations with other variables and usability.

### Internal structure of ANTS, Oxford NOTECHS and OSCAR

#### Internal consistency

Cronbach alpha scores for all categories with all raters combined were good for all tools. However, when raters were considered separately the assessment tool with the lowest score for internal consistency was ANTS (this was even more obvious when categories were considered separately). This may be because less time was taken to consider how each of the raters used ANTS as it was the tool use most frequently. The Civil Aviation Authority [6] requires that trainers’ performance is regularly reviewed, and these results highlight the importance of a similar approach in healthcare settings.

#### Interrater reliability

Measurements of particular attributes in the same subjects may vary greatly between raters and this source of unpredictability is an obvious concern in both clinical settings and high stakes examinations. This is further complicated because many measurements between raters ignore the presence of rater variance and assume that differences are caused by a change in the attribute being assessed, whether that is a clinical sign or a behavior [30].

The challenge in comparing reliability of NTS assessment tools in healthcare is magnified by the variety of different scores analyzed (e.g., means, raw or global scores) and statistical tests used by developers. Most studies of NTS assessment tools use ICC or kappa (usually weighted) but a few use r_WG_ or generalizability theory [8]. The choice of statistical assessment in this study was governed by relevant literature [24, 30, 31]; statistical advice and by tests which had been used in the original studies. Two tests (ICC and weighted kappa) were chosen to analyze the same data and provided an opportunity to highlight the ease with which reliability may be misinterpreted.

This study showed that the ICC scores of three expert raters using three different NTS assessment tools for the analysis of 10 standardized videos ranged from poor (task management in ANTS and SA in Oxford NOTECHS) to very good (problem solving in Oxford NOTECHS and cooperation and SA in OSCAR). ICC is recommended as the test to use for IRR by Gwet [30] (personal communication: “I always first recommend the use of ICC with quantitative (i.e., numeric) measurements regardless of the number of judges”) and Downing [24] and ICC results were good to very good for Raters 1 and 3 in 9 of the 15 categories and all the global scores. However, the weighted kappa results showed only fair agreement in 7 of the 15 categories and moderate agreement for the global scores for all tools.

The IRR for ANTS was surprisingly moderate despite the calibration session prior to rating the videos individually. We were not as explicit about assessing particular elements with ANTS as we were with Oxford NOTECHS and OSCAR because all raters were formally trained in the use of ANTS. Furthermore, whilst each of the three raters is regularly using ANTS in their debriefing sessions they do not routinely do so together and do not formally score participants.

IRR was better for OSCAR than for Oxford NOTECHS (when assessed with ICC) which came as a surprise because Oxford NOTECHS is more similar in structure to ANTS. OSCAR, however, provides more explicit example behaviors (because it is only considering NTS in one clinical situation: cardiac arrest) within the categories which may have reduced variance between raters.

Some authors have recommended generalizability theory (“a statistical framework for examining… the reliability of various observations or ratings” [32]) as the most comprehensive assessment of sources of variance in studies of reliability [24, 33, 34]. However, generalizability theory requires substantial numbers of raters and subjects, and our study was not large enough to produce meaningful results.

Finally, IRR scores for the SA category were very good for ANTS and even better for OSCAR but poor for Oxford NOTECHS. Several of the studies describing NTS tools reference SA as being a challenging category to score [13, 35–37]. It is possible that, in this study, our familiarity with ANTS and the prescriptiveness of OSCAR led to better scores. The lower score for Oxford NOTECHS may relate to the difficulty reported with the scoring system in the qualitative analysis (see Additional file 3).

### Response process

The three raters in this study are all accustomed to using ANTS in formative debriefing settings and rarely ascribe numerical scores to candidates or teams. Performance is considered in the context of what has just played out in the simulator and debriefing uses verbal descriptors and objective examples (either remembered or recorded) of performance to enhance learning in a supportive environment [38, 39]. Ratings of NTS where there is more than one member of faculty are usually derived for categories and global scores by consensus, prior to the debrief beginning and interrater reliability scores are not relevant because complete agreement is reached. Whilst providing a score as a marker of performance is important in discriminating between levels of performance and as a means of calculating IRR, Flin et al. [40] do not recommend the use of scores for formative debriefing.

The assessment tools used in this study all provided different scoring systems. The score range was lowest for ANTS (1–4) and highest for Oxford NOTECHS (1–8) and a ceiling effect was apparent in rater 2’s scores for ANTS. This lack of variance for scores in ANTS may have affected the IRR results. We scored the same scenario (with different candidates) for each of the tools to provide some standardization of expected actions and behaviors. It is interesting to note that both tools which used videos in the original studies to test IRR (ANTS and OSCAR) did not do this.

Much of our data was not normally distributed which is why median values have been displayed as a measure of the central tendency of the scores for each rater. All the original papers discuss mean values with no mention of the distribution of their scores. This is important when one considers that the research group who designed Oxford NOTECHS originally described a 6-point rating scale [12] but later adapted it because it did not allow enough discrimination between candidates or teams. However, the revised score range for Oxford NOTECHS II (1–8) suggests a starting point of 6 (assuming most teams will perform to an acceptable level) which automatically skews scores to the top end of the scale.

### Relations with other variables

Evidence for relations with other variables refers to the “statistical associations between assessment scores and another measure or feature that has a specified theoretical relationship” [9]. The expectation for the three assessment tools in this study is that the relationship between them would be strongly positive as they measure the same construct. The results from this study have revealed that this is the case for Raters 1 and 3 but not Rater 2. This might be explained by the lack of time and experience using the novel tools or by the differences in structure of the response process and different language used to describe categories. There are no data in the original development papers for ANTS and OSCAR to describe relations with other variables and limited data for Oxford NOTECHS (comparison between Oxford NOTECHS and surgical error rates revealed a weak negative association but between Oxford NOTECHS and the Observational Teamwork Assessment for Surgery there was a significant positive correlation [12]). Variability in the evidence for relations with other variables has also been found for NTS assessment tools in other studies [27, 41, 42].

### Usability of ANTS, Oxford NOTECHS and OSCAR

#### Training to use NTS assessment tools

The issue of training to ensure adequate IRR has been raised by several authors [43–46]. The designers of ANTS [13] have designed a two-day bespoke course complete with handbook whereas the authors of Oxford NOTECHS [12] state “the scale can also be used by an observer from a variety of backgrounds, with a small provision for training” and OSCAR [11] “the user would require some limited instruction in its use.” In this study we undertook the training suggested by the authors and found that we did not achieve excellent reliability. Russ et al. [47] describe using 8–10 videos to achieve satisfactory reliability for novice assessors and Spanager et al. [48] found that experienced trainers could achieve good reliability with five. However, in a larger study Graham et al. [49] found that reliability was moderate to poor for a group of experienced anesthetists trained to use ANTS in 1 day, which is more in line with our findings. Our less than perfect agreement may be explained in part by the lack of time spent recalibrating for ANTS and not enough time to train for Oxford NOTECHS and OSCAR, although all raters felt that the training and material were adequate. Patey et al. [50] highlight the importance of training and refreshing skills in NTS assessment, and that substantial barriers exist for educators in healthcare in accessing the necessary training.

#### Quantitative assessment of usability

Other studies have used completeness of score sheets as a marker of usability of a system [20, 51, 52] but this only provides superficial information. The 100% completion rate in this study masked the underlying issues with the tools which were elucidated in the qualitative data.

There was no significant difference between time taken to use Oxford NOTECHS and OSCAR even though OSCAR had more categories and elements to assess. This may be explained by the fact that OSCAR provides explicit guidance for each of its elements, even though the score sheet covers several pages. The shorter time taken to assess with ANTS is explained in part by familiarity with the tool and by the fact that only one team member is being assessed. It would have been interesting to analyze the difference in time taken when all three teams are assessed using Oxford NOTECHS and OSCAR but that was not possible in this study.

#### Qualitative assessment of usability

Whilst statistical evidence of reliability provides useful information about the validity of a tool it does not complete the picture [53]. The analysis of usability highlighted some important differences between the tools which would impact our choice of tool in future studies and are highlighted in Additional file 3. All raters felt that observing behaviors relevant to categories and elements was average to easy with ANTS and Oxford NOTECHS but not with OSCAR and all felt that there were some behaviors missing from OSCAR and that descriptors of behaviors (either good or bad) were not helpful. This may have been because we were only using OSCAR to score the physician group but in our post-study focus group it was clear that the problem stemmed from overlap of behaviors between the physician and anesthesia groups and disagreement about some of the descriptors based on our own clinical experience.

The score sheet for OSCAR caused some challenges in marking videos because it filled several pages requiring the rater to flip between sections when different behaviors were observed. Both the Oxford NOTECHS and OSCAR sheets did not provide enough room for comment which would have been compounded if all teams were being observed. Rating behaviors is also challenging without the necessary context-specific expertise e.g., an anesthetist would have difficulty assessing a surgical scrub-nurse’s behaviors. Both Oxford NOTECHS and OSCAR are designed to be used with “limited instruction” and the original studies showed that IRR was acceptable for raters without clinical experience. Guidance on the use of behaviorally anchored rating scales highlights the need for extensive training in their use (especially for high-stakes settings), that they do not apply across domains and cultures (i.e., aviation to medicine, doctor to nurse) and that understanding of the context of application is vital.

## Study strengths and limitations

The three expert raters in this study are more familiar with the ANTS assessment tool than Oxford NOTECHS or OSCAR, all had undertaken formal training in the use of the tool but no further calibration was undertaken for the study. To mitigate for this, we produced a standardized questionnaire which had been validated for use in the assessment of NTS tools [22] to provide an objective assessment of the different systems.

Measuring the time taken to score the videos by one rater (the only rater not involved in the START study) provided only limited data on usability of the tools. Future studies would aim to collect these data for more raters to improve the value of these results in drawing conclusions about the use of a specific tool.

We assessed the three tools in this study by asking three expert raters to review 10 standardized videos with each tool. Gwet [30] has highlighted that the higher the number of subjects, raters and categories the more likely the output from the agreement statistic is to be accurate and, therefore, meaningful. This study would have benefitted from the use of more raters or a larger sample size, but the design was pragmatic in the context of the time available.

Capturing and recording assessments of NTS in a scenario depicting changes happening over a short period of time is challenging and it is possible that our raters missed or misinterpreted behaviors leading to inaccurate scores [54, 55].

There were three people forming the team in each scenario – two of them were faculty members who were playing the role of additional staff members in the room and whilst this may have detracted from the realism of the situation, they had been primed to respond as they would in real life to the anesthetist leading the management of the cardiac arrest. We used a standardized cardiac arrest scenario where the expected team responses and actions were the same each time in order to reduce the impact of an additional source of variability.

Observer bias may have impacted our results as both Raters 2 and 3 were involved in running the START project. However, the study was completed over 2 years before this analysis and so memory of the scenarios was reduced. Furthermore, we randomized both the videos we chose and the order of viewing when using each tool [56, 57].

Only one of the tools in the study was specifically designed for the measurement of NTS in resuscitation (OSCAR) – the other two, however, were designed for the assessment of NTS in anesthetists in elective and emergency settings (including cardiac arrest). Furthermore, OSCAR and Oxford NOTECHS do not provide the option to record that a behavior was not observed which could lead to a falsely low score in an otherwise highly performing team.

## Conclusion

The results from this study resonate with the challenges faced in analyzing and comparing NTS assessment tools revealed in published systematic reviews [8, 58]. Since the publication of these reviews further NTS assessment tools have been developed for a diverse range of settings including a tool for assessment of medical students [59] and a tool for NTS in cataract surgery [60]. A recent editorial summed up the situation succinctly: (there is) “a vast amount of work yet to do to quantify the impact of NTS in healthcare and standardize assessment. We need more robust data, a parsimonious set of NTS and a set of benchmarks and incentives to guide adoption among clinicians” [61]. In light of the renewed focus on simulation as an educational tool to support training recovery in the aftermath of COVID-19, it is even more important that the way we assess these vital skills is simplified, standardized appropriately, and supported with adequate training.

## Supplementary Information

**Additional file 1.** Score sheets for ANTS, Oxford NOTECHS and OSCAR. Original score sheets for ANTS, Oxford NOTECHS and OSCAR with descriptions of categories, elements and range of scores.

**Additional file 2.** Usability evaluation questionnaire for ANTS, Oxford NOTECHS and OSCAR (adapted with permission from Dr. J Rutherford). Questionnaire consisting of 16 questions formatted either as binary yes/no responses or Likert scales with space for free text comments.

**Additional file 3.** Summary of quantitative and qualitative feedback on use of ANTS, Oxford NOTECHS and OSCAR. Responses to usability questionnaire in Additional file 2. Quantitative data and summarized qualitative data are presented in tables.

## Data Availability

The datasets generated and/or used during this study are available from the corresponding author on reasonable request.

## Abbreviations

ANTS: Anesthetists’ Non-Technical Skills
ANTS-AP: anesthetic non-technical skills for anesthetic practitioners
ICC: Intraclass correlation coefficient
IQR: Interquartile range
NTS: Non-Technical Skills
OSCAR: Observational Skill Based Assessment tool for Resuscitation
Oxford NOTECHS: Oxford Non-Technical Skills tool
SA: Situation awareness
START: ST1 Anesthetic Recruit Training
