# Supplementary material for: A study of validity and usability evidence for non-technical skills assessment tools in simulated adult resuscitation scenarios
Source: BMC Med Educ. 2023 Mar 11;23:153. doi: 10.1186/s12909-023-04108-4 (PMC10007667; doi:10.1186/s12909-023-04108-4)
Supplement: Supplementary file 1 — Additional file 1. Score sheets for ANTS, Oxford NOTECHS and OSCAR. Original score sheets for ANTS, Oxford NOTECHS and OSCAR with descriptions of categories, elements and range of scores. [file 12909_2023_4108_MOESM1_ESM.pdf]

Additional file 1: Score sheets for ANTS, Oxford NOTECHS and OSCAR

ANTS score sheet

Category	Element	*Rating	Observation on Performance	Category rating and debriefing notes
Task Management	Planning & preparing			
	Prioritising			
	Providing & maintaining standards			
	Identifying & utilising resources			
Team Working	Co-ordinating activities with team			
	Exchanging information			
	Using authority & assertiveness			
	Assessing capabilities			
	Supporting others			
Situation Awareness	Gathering information			
	Recognising & understanding			
	Anticipating			
Decision Making	Identifying options			
	Balancing risks & selecting options			
	Re-evaluating			

*4 Good; 3 Acceptable; 2 Marginal; 1 Poor; N Not Observed

15

Oxford NOTECHS score sheet

		Surgeon team	Anesthetic team	Nursing team
Leadership & management	Leadership<br>Maintenance of standards<br>Planning and preparation<br>Workload management<br>Authority & Assertiveness			
Teamwork & cooperation	Team building/maintaining<br>Support of others<br>Understanding team needs<br>Conflict solving			
Problem solving & decision making	Definition & diagnosis<br>Option generation<br>Risk assessment<br>Outcome review			
Situation awareness	Notice<br>Understand<br>Think ahead			

1	2	3	4	5	6	7	8
consistent	inconsistent	consistent	inconsistent	inconsistent	consistent	inconsistent	consistent
Behavior compromises patient safety and effective teamwork		Behavior in other conditions could directly compromise patient safety and effective teamwork		Behavior maintains an effective level of patient safety and teamwork		Behavior enhances patient safety and teamwork, a model for all other teams	

Range 12-96 points

OSCAR score sheet

0 = Team Severely Compromised	1 = Team Compromised
2 = Slight detriment to team	3 = Team neither enhanced or hindered
4 = Moderate enhancement to team	5 = High level of enhancement to team
6 = Highly effective in enhancing teamwork	

COMMUNICATION

Anesthetic Group (A)	Individual Behavior Ratings							Overall Imp (0-6)
Informs team whether patient is making respiratory effort	0	1	2	3	4	5	6	
Informs team of any other relevant clinical signs eg dilated pupil, obvious injuries, signs of aspiration	0	1	2	3	4	5	6	
Communication to team that they plan to intubate the patient if required	0	1	2	3	4	5	6	
Requests patient history on arrival and communicates details to team, if required	0	1	2	3	4	5	6	
**Physician Group (P)**								
Reviews patient history and notes and communicates relevant details clearly to the team	0	1	2	3	4	5	6	
Clear instructions communicated to the team regarding the arrest protocol	0	1	2	3	4	5	6	
Encourages communication from sub-teams, and encourages team members to give opinions	0	1	2	3	4	5	6	
**Nurse Group (N)**								
Provides clear information about arrest events on arrival of arrest team	0	1	2	3	4	5	6	
Senior nurse provides clear, audible requests to junior nurse when requesting equipment e.g. additional iv bags	0	1	2	3	4	5	6	
Instructs other nurses on ward clearly how to assist with arrest or other ward duties as appropriate	0	1	2	3	4	5	6	

CO-OPERATION

Anesthetic Group (A)	Individual Behavior Ratings							Overall Imp (0-6)
A-group provides information on request from M-group (e.g. about the airway)	0	1	2	3	4	5	6	
A-group assists M-group in decision making in difficult scenarios	0	1	2	3	4	5	6	
**Physician Group (P)**								
Responds to questions from other team members about decisions made regarding the arrest	0	1	2	3	4	5	6	
Supports less experienced members of M-group, and compensates for their lack of experience	0	1	2	3	4	5	6	
**Nurse Group (N)**								
Provide support and assistance to A-group and M-group when needed eg finding airway adjuncts	0	1	2	3	4	5	6	
Help M-group locate items not routinely stocked on trolley, or missing from the trolley	0	1	2	3	4	5	6	
Assist M-group with extra tasks e.g. sending bloods, contacting family, contacting labs etc	0	1	2	3	4	5	6	

CO-ORDINATION

Anesthetic Group (A)	Individual Behavior Ratings							Overall Imp (0-6)
Information provided about changes in patient condition as they occur	0	1	2	3	4	5	6	
A-group co-ordinate team to move patient eg floor to bed, up bed	0	1	2	3	4	5	6	
**Physician Group (P)**								
Notifies N and A groups of anticipated further requirements for patient resuscitation	0	1	2	3	4	5	6	
Within M group, co-ordinates tasks such as taking of bloods, sending samples, sending ABG etc	0	1	2	3	4	5	6	
**Nurse Group (N)**								
Prepare Resus Trolley for use by team by bringing to bedside, turning monitor on etc	0	1	2	3	4	5	6	
Prepare further drugs in readiness for their next required use e.g. prepare next adrenaline minijet	0	1	2	3	4	5	6	
A Senior Nurse (Sister) is always present to provide backup to Staff Nurse	0	1	2	3	4	5	6	

LEADERSHIP

Anesthetic Group (A)	Individual Behavior Ratings							Overall Imp (0-6)
Advises team on best management, and contingency plans for patient, and takes lead if required	0	1	2	3	4	5	6	
Anaesthetist assertively takes a lead in Airway control and Ventilation on arrival at arrest	0	1	2	3	4	5	6	
Lead Anaesthetist supervises and supports staff lacking familiarity with tasks or equipment	0	1	2	3	4	5	6	
**Physician Group (P)**								
Takes a lead and clearly instructs assistants with requirements for arrest and/or defers leadership as required if appropriate	0	1	2	3	4	5	6	
Supervision given to staff lacking experience or familiarity with tasks or equipment	0	1	2	3	4	5	6	
Instructs N-group of additional requirements e.g. recent blood results from computer, to call the family	0	1	2	3	4	5	6	
**Nurse Group (N)**								
Takes a lead with initial Basic Life Support attempts until Arrest Team arrive	0	1	2	3	4	5	6	
Supervision and support given to junior or inexperienced members of N-team	0	1	2	3	4	5	6	

26 **MONITORING**

Anesthetic Group (A)	Individual Behavior Ratings							Overall Imp (0-6)
Maintains monitoring of patient condition, signs of respiration, other clinical signs	0	1	2	3	4	5	6	
Checks ventilation is adequate with regular blood gas analysis and amends ventilation accordingly	0	1	2	3	4	5	6	
Confirms drug identity by checking syringe labeling prior to drug administration	0	1	2	3	4	5	6	
**Physician Group (P)**								
Maintains awareness of activities of other teams e.g. anaesthetist intubating	0	1	2	3	4	5	6	
Monitors progress of resuscitation protocol with careful checking of time, and constant reassessment of limb of protocol and “extra considerations”	0	1	2	3	4	5	6	
Checks team condition e.g. monitors for fatigue in team members from CPR and suggests team members change roles, take turns etc	0	1	2	3	4	5	6	
**Nurse Group (N)**								
Monitors patient dignity and considers well-being of other patients nearby	0	1	2	3	4	5	6	
Maintains awareness of the needs of M and A groups	0	1	2	3	4	5	6	

27 **DECISION MAKING**

Anesthetic Group (A)	Individual Behavior Ratings							Overall Imp (0-6)
Prompt identification of the problem	0	1	2	3	4	5	6	
Rapidly and clearly outlines a strategy or plan, and asks for equipment	0	1	2	3	4	5	6	
Anticipates potential problems and prepares accordingly – e.g. asks for further blood crossmatched	0	1	2	3	4	5	6	
**Physician Group (P)**								
Rapidly decides an appropriate course of action for continued resuscitation	0	1	2	3	4	5	6	
Uses the team as a whole to help develop options – asks for opinions and processes them decisively	0	1	2	3	4	5	6	
**Nurse Group (N)**								
Prompt decision making during initial resuscitation attempts	0	1	2	3	4	5	6	
Anticipates potential problems A and M teams may encounter e.g. pulls bed out from wall, clears area etc	0	1	2	3	4	5	6	
Appropriate decision making regarding timing of initial decision to put out a cardiac arrest call	0	1	2	3	4	5	6	

28
