# Supplementary material for: A study of validity and usability evidence for non-technical skills assessment tools in simulated adult resuscitation scenarios
Source: BMC Med Educ. 2023 Mar 11;23:153. doi: 10.1186/s12909-023-04108-4 (PMC10007667; doi:10.1186/s12909-023-04108-4)
Supplement: Supplementary file 2 — Additional file 2. Usability evaluation questionnaire for ANTS, Oxford NOTECHS and OSCAR (adapted with permission from Dr. J Rutherford). Questionnaire consisting of 16 questions formatted either as binary yes/no responses or Likert scales with space for free text comments. [file 12909_2023_4108_MOESM2_ESM.pdf]

Additional file 2: Usability evaluation questionnaire for ANTS, Oxford NOTECHS and OSCAR (adapted with permission from Dr J Rutherford)

General questions about the system (Questions were applied to all tools except where indicated)

1. Do you think the system was useful for structuring your observation of the film scenarios?

Yes / No If no, what was the problem?

2. Did it seem to address the key non-technical skill behaviors displayed by the individuals/team in the scenario?

Yes / No If no, what behaviors do you think were not addressed?

3. How easy was it to associate observed behaviors with the NTS tool's categories?

Very difficult / Difficult / Average / Easy / Very easy Please provide any specific comments about any or all of the categories:

4. Do you think there are any (non-technical) skills elements and/or categories missing from the list?

Yes / No If yes, what is missing?

5. Do you think there are any (non-technical) skills elements and/or categories in the list which are not necessary?

Yes / No If yes, which elements and/or categories are unnecessary?

6. Was the wording used for the category and element labels meaningful?

Yes / No If no, please describe where you thought there were problems

7. Were the descriptions for each category and element clear?

Yes / No If no, please describe which descriptions were unclear

8. Were the examples of 'good' behaviors helpful?

Yes / No Please give any comments (positive or negative) you may have

9. Were the examples of 'poor' behaviors helpful?

Yes / No Please give any comments (positive or negative) you may have

Questions about the rating scale

10. Please indicate how easy it was to use the rating scale provided:

Very difficult / Difficult / Average / Easy / Very easy

If you have any particular concerns please explain

11. Do you think the rating scale gave you enough flexibility to rate the performance levels seen in the film clips?

Yes / No

If no, would you have liked a longer or shorter scale?

longer / shorter

12. Did you use the comments section on the rating form?

Yes No

If yes, please say what sort of information you noted down e.g. explanation of the performance rating you gave

13. Did you have any problems with the design of the rating form?

Yes / No

If yes, please explain

14. Was the amount of background information you were given (ANTS excluded):

too much / just right / too little

15. Were the explanations of the different categories and behavioral markers adequate (ANTS excluded)?

Yes / No

If no, how do you think the explanations could be improved?

16. Overall do you think you were able to use the NTS system effectively?

Yes / No

<u>Any other comments?</u>
