# Supplementary material for: A study of validity and usability evidence for non-technical skills assessment tools in simulated adult resuscitation scenarios
Source: BMC Med Educ. 2023 Mar 11;23:153. doi: 10.1186/s12909-023-04108-4 (PMC10007667; doi:10.1186/s12909-023-04108-4)
Supplement: Supplementary file 3 — Additional file 3. Summary of quantitative and qualitative feedback on use of ANTS, Oxford NOTECHS and OSCAR. Responses to usability questionnaire in Additional file 2. Quantitative data and summarized qualitative data are presented in tables. [file 12909_2023_4108_MOESM3_ESM.pdf]

Additional file 3: Summary of quantitative and qualitative feedback on use of ANTS, Oxford NOTECHS and OSCAR

Question	1	2	3	4	5	6	7	8	9	10	11	12	13	14	15	16
**ANTS**																
**Rater 1**	Y	Y	Easy	N	N	Y	Y	Y	Y	Very easy	Y	Y	N	N/A	N/A	Y
**Rater 2**	Y	Y	Average	N	N	Y	Y	Y	Y	Easy	Y	N	N	N/A	N/A	Y
**Rater 3**	Y	Y	Easy	N	N	Y	Y	Y	Y	Easy	Y	Y	N	N/A	N/A	Y
**Oxford NOTECHS**																
**Rater 1**	Y	Y	Easy	N	N	Y	Y	Y	Y	Easy	Y	Y	Y	Just right	Y	Y
**Rater 2**	Y	Y	Easy	N	N	Y	Y	Y	Y	Very easy	Y	N	N	Just right	Y	Y
**Rater 3**	Y	Y	Easy	N	N	Y	Y	Y	Y	Easy	Y	N	Y	Too much	Y	Y
**OSCAR**																
**Rater 1**	N	Y	Average	Y	N	Y	Y	N	N	Difficult	N	Y	Y	Just right	N	N
**Rater 2**	N	Y	Difficult	Y	N	Y	Y	N	Y	Very difficult	Y	N	Y	Too little	N	N
**Rater 3**	N	Y	Difficult	Y	N	N	Y	N	N	Difficult	N	N	Y	Just right	Y	N

Questions:

1. Do you think the system was useful for structuring your observation of the film scenarios?
2. Did it seem to address the key non-technical skill behaviors displayed by the individuals/team in the scenario?
3. How easy was it to associate observed behaviors with the NTS tool's categories?
4. Do you think there are any (non-technical) skills elements and/or categories missing from the list?
5. Do you think there are any (non-technical) skills elements and/or categories in the list which are not necessary?
6. Was the wording used for the category and element labels meaningful?
7. Were the descriptions for each category and element clear?
8. Were the examples of 'good' behaviors helpful?
9. Were the examples of 'poor' behaviors helpful?
10. Please indicate how easy it was to use the rating scale provided:
11. Do you think the rating scale gave you enough flexibility to rate the performance levels seen in the film clips?
12. Did you use the comments section on the rating form?
13. Did you have any problems with the design of the rating form?
14. Was the amount of background information you were given (ANTS excluded):
15. Were the explanations of the different categories and behavioral markers adequate (ANTS excluded)?
16. overall do you think you were able to use the NTS system effectively?

3 Summary of qualitative feedback

Comments about the systems overall		
**ANTS**	ADVANTAGES:<br>The category and element descriptors are described in general terms making them easy to apply across all situations<br>There is a comprehensive handbook with useful summary pages	DISADVANTAGES:<br>Only anesthetist in the team assessed – designed specifically for this purpose<br>Some behaviors cross categories leading to difficulty in determining where to ascribe a score<br>It is recommended that faculty undertake a two-day training course in the use of the system
**Oxford NOTECHS**	ADVANTAGES:<br>Assesses three sub-teams in theatre (surgeons, the anesthetic team and the surgical scrub team) and provides scores for each of these sub-teams<br>Provides a global team score<br>NTS domains are limited to 4 increasing ease of use<br>Structure of NTS domains similar to ANTS (making it a more readily usable than OSCAR)	DISADVANTAGES:<br>Would require additional faculty with context specific expertise to use whole tool
**OSCAR**	ADVANTAGES:<br>OSCAR allows a comprehensive assessment of NTS in three sub-teams during the management of arrest situations<br>Examples of behaviors given are specific and comprehensive which may help when faculty are new to using the tool	DISADVANTAGES:<br>Example behaviors quite prescriptive – if they don't happen an otherwise well performing team may be inappropriately marked down<br>6 domains are challenging to mark – might be helpful to combine e.g. co-operation and co-ordination<br>Blurring of lines between roles of Anesthetic and Physician group could make it difficult to score if not experienced in resuscitation<br>Some example behaviors did not fit with the authors' experience of role responsibilities at an arrest e.g. overlap in decision making between Anesthetic group and Physician group and clarity on who is leading in an arrest
**Comments about the rating scale**		
**ANTS**	ADVANTAGES:<br>Provides option of marking a behavior "not observed"<br>Scoring system on one page with room for notes	DISADVANTAGES:<br>Only scoring anesthetic NTS<br>Problem with ceiling effect using 1-4 scale<br>Does not provide a global rating
**Oxford NOTECHS**	ADVANTAGES:<br>Scoring system all on one page<br>Less risk of ceiling effect with 1-8 scale<br>Descriptors provided for each domain	DISADVANTAGES:<br>Not enough space to make notes on score sheet<br>Suggested that a starting point of 6 for each assessment but automatically biases to higher end of scale - points 1-5 describe sub-optimal behavior<br>The descriptors "consistent" and "inconsistent" were challenging to use in some scenarios<br>No option to mark a behavior as "not observed"

**OSCAR**	ADVANTAGES:<br>Score sheet is comprehensive with descriptors for each behavior under consideration<br>Less risk of ceiling effect with 1-6 scale	DISADVANTAGES:<br>Not much distinction between a score of 5 (high level of enhancement to team) and a score of 6 (highly effective in enhancing teamwork)<br>No option to mark a behavior as “not observed”<br>Score sheet covers 3 pages making it challenging to move back and forward between domains during marking period<br>Scoring system covers three professional groups - would necessitate additional faculty<br>More space to make notes would be good

4
